# The data of Isobaric tags for relative and absolute quantification-based proteomic analysis of defense responses triggered by the fungal pathogen *Fusarium graminearum* in wheat

**DOI:** 10.1016/j.dib.2019.104747

**Published:** 2019-11-02

**Authors:** Biao Wang, Xuefeng Li, Wuying Chen, Lingrang Kong

**Affiliations:** State Key Laboratory of Crop Biology, Shandong Key Laboratory of Crop Biology, College of Agronomy, Shandong Agricultural University, Taian, 271018, China

**Keywords:** Fusarium head blight, Resistance, Proteomics, iTRAQ, Protein mass

## Abstract

Fusarium head blight (FHB) is one of the most prevalent diseases of wheat and other small grain cereals that is predominantly caused by the fungal pathogen *Fusarium graminearum*. Extraction of total proteins were from tissues of A061-3 and A061-4 plants. Three biological replicates were carried out for each line at four time points. Samples were performed using iTRAQ (Isobaric tags for relative and absolute quantification). This data is being made available to increase the understanding of FHB resistance proteomics. The data from this study are related to the research article “Isobaric tags for relative and absolute quantification-based proteomic analysis of defense responses triggered by the fungal pathogen *Fusarium graminearum* in wheat” [1].

**Table:** Specifications Table

Subject	Agricultural and Biological Sciences (Agronomy and Crop Science)
Specific subject area	Wheat proteomics
Type of data	Figures
How data were acquired	Mass spectrometry analysis, Nano HPLC-Q Exactive system (Thermo Scientific), using the RIGOL L-3000 HPLC Pump system.
Data format	Raw and Analyzed
Parameters for data collection	Proteins were extracted from wheat panials
Description of data collection	Bioinformatics analysis contained the Hierarchical clustering, Gene Ontology annotation, Kyoto Encyclopedia of Genes and Genomes, Venn diagrams.
Data source location	Shandong Agricultural University , Taian city , China Samples were collected from the greenhouse (36°13′N, 117°13′E) at Shandong Agricultural University, Taian city.
Data accessibility	With the article Repository name: Mendeley Data Data identification number: DOI: 10.17632/45c3dgg68n.1 Direct URL to data: https://data.mendeley.com/datasets/45c3dgg68n/1
Related research article	The data article was associated with the research article [1]. Biao Wang, Xuefeng Li, Wuying Chen, Lingrang Kong. Isobaric tags for relative and absolute quantification-based proteomic analysis of defense responses triggered by the fungal pathogen Fusarium graminearum in wheat. Journal of Proteomics

**Table:** 

**Value of the Data** • This data extends iTRAQ method for wheat panials from two near-isogenic lines for the first time. • The data contributed to the distribution of proteins species involved in the FHB resistance for experts of wheat breeder. • The data provided information about reference protein species data for further genetic studies and could be useful for comparative studies FHB resistance.

## 1 Data

**Fig. 1:**
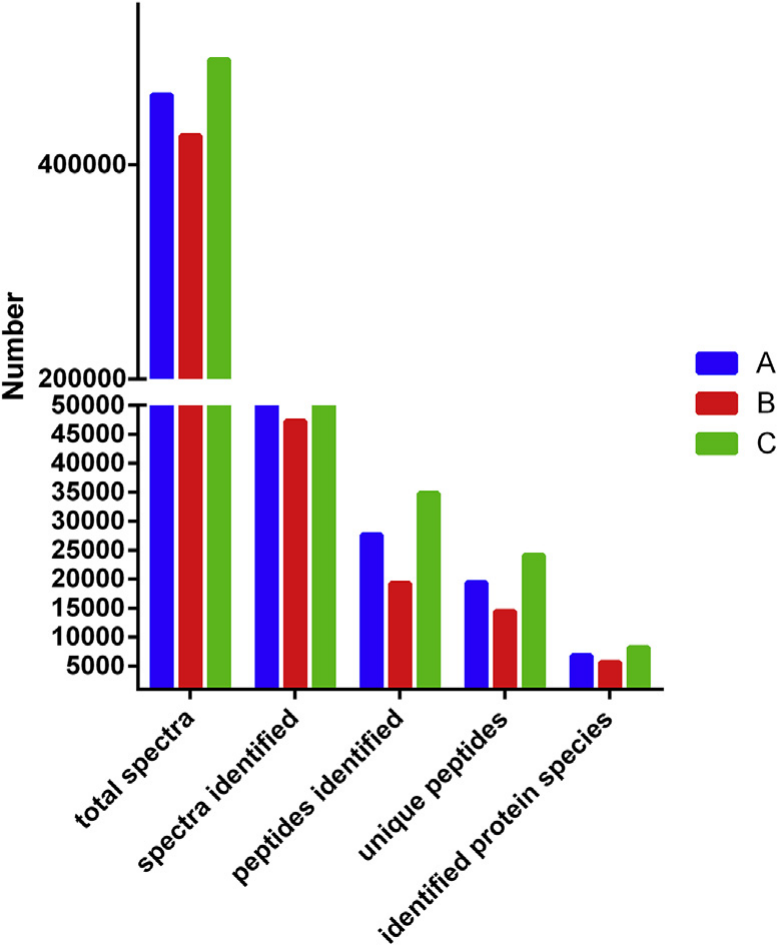
The number of the protein species information.

**Fig. 2:**
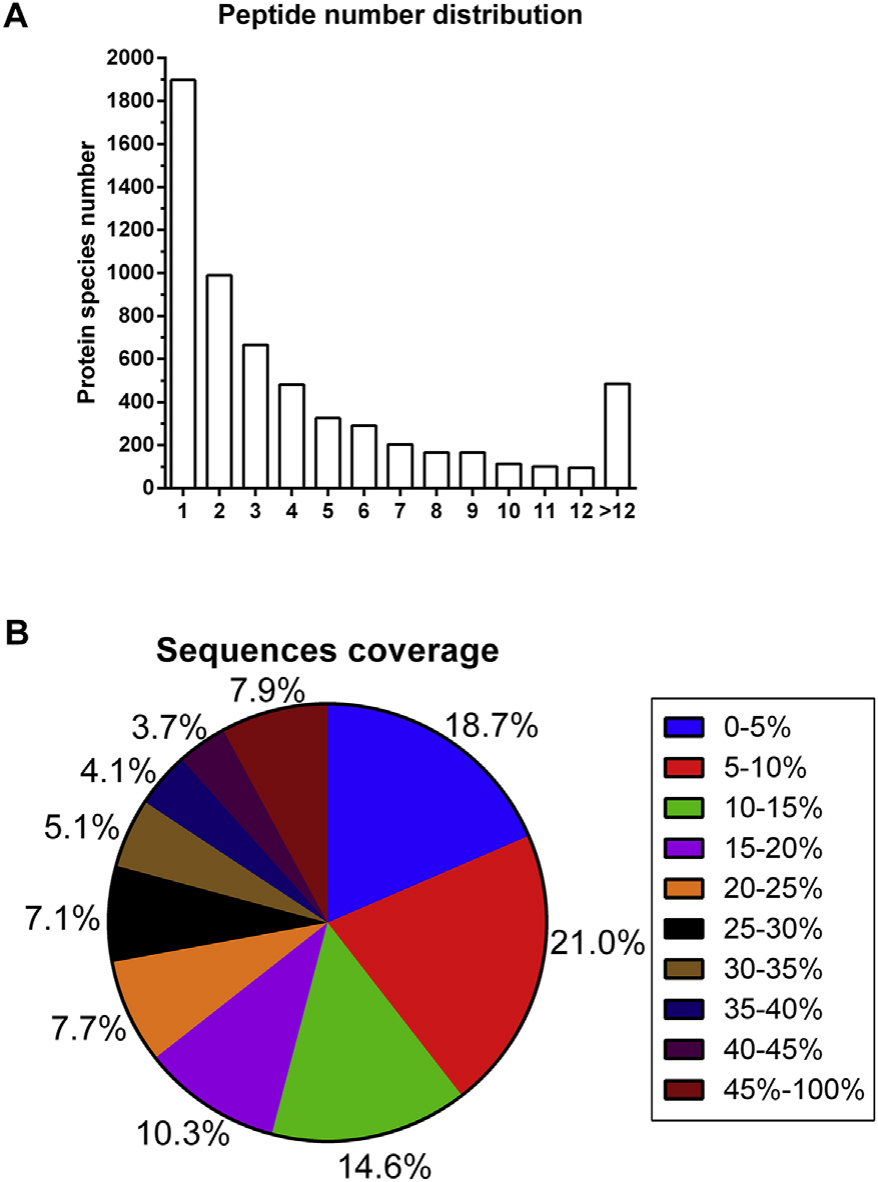
The number of peptide distribution.

**Fig. 3:**
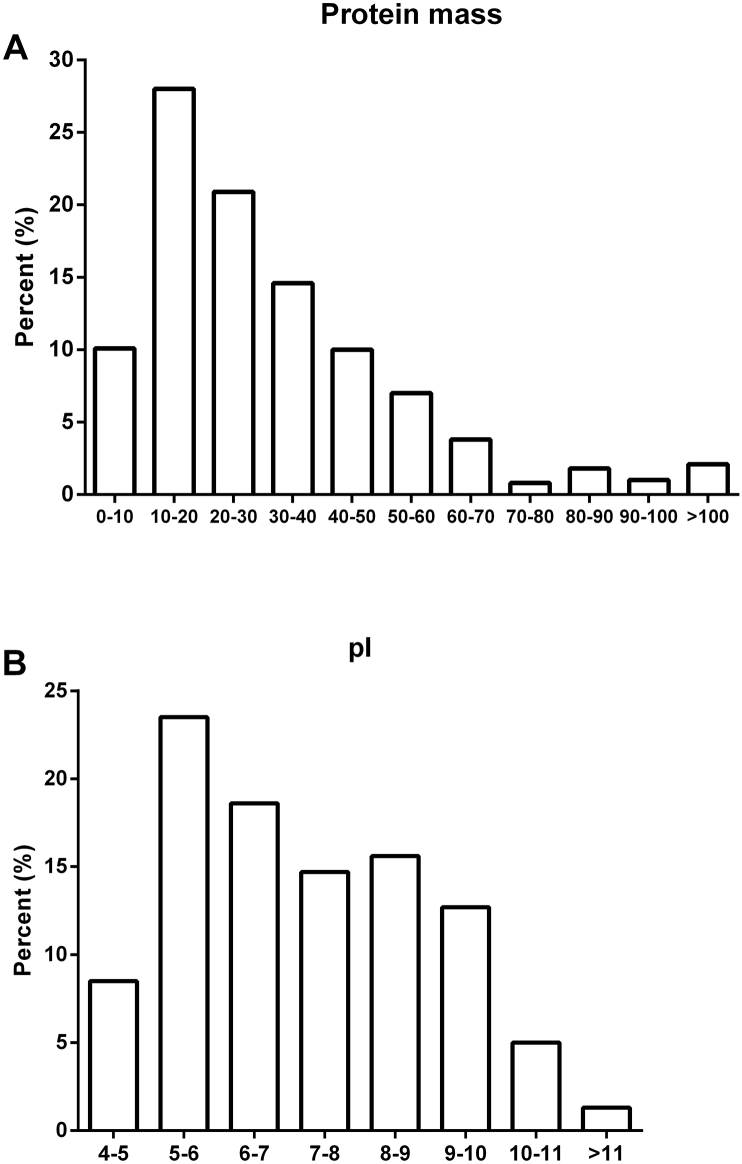
Protein mass and pI of differentially accumulated proteins.

Here we report the proteomic analysis of excretion from two lines of wheat. In study, total spectra of three biological replicates included more than 465,392, and identified protein species in three biological replicates were 6,797, 5,653, 8,208, respectively (Fig. 1 and supplementary Table S1). The distribution of the number of peptides defining each protein showed that most 77% of the proteins contained at least two peptides (Fig. 2A and supplementary Table S2). Protein sequences with coverage of 0–5%, 5–10%, 10–15%, 15–20%, 20–25%, 25–30%, 30–35%, 35–40%, 40–45%, 45–100% accounted for 14.9%, 14.5%, 14.7%, 11.6%, 9.3%, 7.5%, 6.4%, 5.6%, 4.4%, 11.0%, respectively (Fig. 2B and supplementary Table S2). Further, the samples were performed by multiple enzymatic digestion for the enhancement in sequence coverage. Most identified proteins shared molecular mass of 0–70 kDa, the molecular mass of 10.1%, 28.0%, 20.9%, 14.6%, 10.0%, 7.0%, 3.8% of identified gene products were 0–10, 10–20, 20–30, 30–40, 40–50, 50–60, 60–70, respectively (Fig. 3 and supplementary Table S3). In additional, protein mass of 2.1% of identified proteins species was >100 kDa and there were about 3.6% of identified proteins species during 70–100 kDa (Fig. 3A and supplementary Table S3). Peptides with pI values in the range of 5–6 included 23.5% of identified proteins, which was the maximum of identified proteins, and most of proteins contained (Fig. 3B and supplementary Table S3). Supplementary Tables present the raw data. Supplementary Table S1 present the raw data of protein species in three replicates. Supplementary Table S2 present the raw data for the distribution of peptides and protein sequences with coverage. Supplementary Table S3 present the raw data for the distribution of protein mass and peptides with pI value.

## 2 Experimental design, materials, and methods

1. Preparation of samples. The sample was dissolved in lysis buffer. The concentration of the extracted protein was measured by the Bradford protein assay [2].
2. Trypsin digestion, iTRAQ labelling, high pH reversed-phase HPLC, quantitative proteome analysis by LC-MS/MS, data analysis were performed generally according to Wang et al. [3]. The procedure will be reported in detail by Wang et al. (Journal MethodX, title of “Development of methodologies for the Isobaric Tag for Relative and Absolute Quantification in wheat panials).
